# Detection of left ventricular thrombus in newly diagnosed heart failure patients: diagnostic accuracy of 2D transthoracic echocardiography compared with cardiac MRI in a Kenyan cohort

**DOI:** 10.1186/s44156-025-00092-1

**Published:** 2025-11-03

**Authors:** Barbara Karau, Nadeem Kassam, Mohamed Varwani, Mohamed Jeilan, Kevin Ombati, James Orwa, Mzee Ngunga

**Affiliations:** 1 Department of Internal Medicine, Aga Khan University Hospital, Nairobi, Kenya; 2 Department of Radiology, Aga Khan University Hospital, Nairobi, Kenya; 3 Department of Population health, Aga Khan University Hospital, Nairobi, Kenya

**Keywords:** Heart failure, LV thrombus, Comparison, TTE, CMR, Kenya

## Abstract

**Background:**

Left ventricular thrombus (LVT) is a recognized complication of severe left ventricular (LV) systolic dysfunction. While cardiac magnetic resonance (CMR) Imaging is considered the gold standard due to its high sensitivity and specificity for detecting LVT, its availability remains limited in Sub-Saharan Africa. In contrast, 2D transthoracic echocardiography (TTE) is more accessible and commonly used in clinical practice. This study aimed to determine the incidence of LVT in patients with a left ventricular ejection fraction (LVEF) of less than 35% from any etiology, and to compare the diagnostic accuracy of TTE against CMR for its detection.

**Methods:**

This prospective, cross-sectional study was conducted at the Aga Khan University Hospital Nairobi (AKUH, N). The study included patients aged 18 years and above with new onset heart failure symptoms and severely reduced LVEF of ≤ 35%. The study was conducted between January 2021 and December 2023. Patients underwent non-contrast 2D TTE and CMR within a 14-day interval. Diagnostic accuracy of 2D TTE was assessed using sensitivity, specificity, and predictive values.

**Results:**

A total of 100 patients were included in the final analysis. The median age of participants was 58.0 years (IQR: 47.0–67.0). The median LVEF was 30% (IQR: 20–33). The median time between 2D echocardiography and cardiac MRI was 10 days (IQR: 1–12). LVT was detected in 11 patients (11%) on CMR. A significantly higher proportion of those with LVT had an ischemic etiology (*n* = 10, 90.9%) (*p* < 0.05). Participants with LVT had a lower LVEF (19%, IQR: 14–31) and larger left ventricular internal diameters (IQR 62 mm, IQR: 55–77.5) (p- value < 0.05). Among those with LVT, 73% (*n* = 8) had it detected by both diagnostic modalities. The 2D TTE’s sensitivity and specificity for detecting LVT were 0.72 and 0.78, respectively, with an excellent negative predictive value of 0.98.

**Conclusion:**

11% of patients were found to have LVT on CMR. Patients with ischemic cardiomyopathy, significantly reduced LVEF, and a dilated LV, were identified as being at the highest risk for developing LVT. 2D TTE showed an excellent negative predictive value in excluding the presence of LVT.

## Introduction

LVT is a life-threatening complication in patients with systolic heart failure, particularly those with moderate to severely reduced LV ejection fraction [1, 2]. It is often linked to large acute anterior myocardial infarctions (MI) and severe forms of dilated cardiomyopathy (DCM) [1, 2]. Detecting LVT is crucial for clinical outcomes and treatment decisions, as it serves as a source of systemic embolization [3]. Two imaging techniques typically used to identify LVT are 2D TTE and CMR [4]. While 2D TTE, which is operator-dependent, widely accessible and relatively affordable in our region, CMR is considered the gold standard due to its superior spatial resolution and tissue characterization. However, CMR’s limited availability makes it an impractical option in many low- and middle-income countries (LMICs), including the Sub-Saharan region [4].

To date, there have been few comparative studies demonstrating the diagnostic accuracy of 2D TTE vs. CMR for the detection of LVT with varying results [5–7]. Nonetheless, the majority of the aforementioned have demonstrated CMR’s superior accuracy. Delayed Enhancement (DE) CMR, a validated technique used to assess the viability of the myocardium is also used in identifying LVT which manifests as an absence of the gadolinium uptake due to the thrombus avascular composition [6, 8]. Despite the diagnostic superiority of CMR, its high cost, limited availability, lack of human resource in performing and interpreting CMR studies in Kenya, all hinder the routine usage of this diagnostic modality for our population [9].

This prospective study was conducted to assess the prevalence of LVT using DE CMR imaging in patients with significantly reduced LV ejection fraction and to evaluate the diagnostic accuracy of 2D TTE in detecting LVT.

## Methodology

This study was a prospective, cross-sectional analytical research conducted at AKUH, N, one of the few hospitals in the region accredited by the Joint Commission International (JCI). This accreditation affirms the hospital’s recognition as a global leader in healthcare.

### Study population and procedure

The population consisted of patients aged 18 years and above with new onset heart failure symptoms having a reduced LVEF of 35% and less (High- risk population for LVT) on 2D TEE at laboratory of AKUHN between January 2021 and December 2023, thought it was temporarily interrupted during the COVID − 19 pandemic. Patients were enrolled if 2D TEE and CMR were done within the 14-day interval. Patients unable to undergo both imaging modalities within 14 days, having extreme claustrophobia, having an implanted MRI incompatible device, those with risk of developing nephrogenic systemic fibrosis or worsening kidney dysfunction and having a previous diagnosis of LVT were excluded from the study design. Imaging was conducted for all patients following a predefined protocol and interpreted by assigned readers who were blinded to the clinical history and results of other imaging modalities.

## Imaging protocol

- **Echocardiography procedure**.

Non-contrast 2D TTE images were acquired in standard planes according to the latest American society of echocardiography guidelines [10]. 2D TTE was performed using standard commercial equipment (Philips IE33Amsterdam, The Vivid 7 General electric Fairfield, CT, USA) by experienced echo-technicians. Post processing evaluation was done using Echo PAC (General Electric) and the findings were reported by a consultant cardiologist. Assessment of LVEF was done in accordance to the 2015 American Society of Echocardiography and the European Association for Cardiovascular Imaging (ASE/EACVI) guideline [11]. LV volumes and Ejection fraction were measured using Simpson’s Biplane method of discs. Left Atrial Volume Index (LAVI) was measured with an area-length formula in Biplane, indexed to BSA. LV mass was calculated from linear measurements and indexed to body surface area (BSA). Right ventricular systolic function was assessed using Tricuspid Annular Plane Systolic Excursion (TAPSE). Tricuspid regurgitation velocity was measured by applying Continuous Wave (CW) Doppler across the tricuspid valve. Left ventricular Diastolic function was assessed using Mitral Inflow pulse wave Doppler and Left ventricular tissue velocity in early diastolic (e’) and were measured at the septal and lateral border of the mitral plane. Wall motion score (WMS) was calculated using the American Society of Echocardiography 17 segment model [10].

- **CMR procedure**.

ECG gated CMR with breath-holding instructions was performed using a 3 Tesla scanner (Philips Ingenia). Cine-CMR was performed initially in 2, 3 and 4 chamber orientations using a balanced Fast Field sequencing. Short axis stacks were acquired from the level of the mitral annulus to the apex for functional analysis. Gadolinium was administered (0.2 mmol/kg) and DE-CMR was performed 10 min later using an inversion recovery pulse sequence using the same chamber orientations as the cine images. Wall motion score (WMS) was calculated using the American Society of Echocardiography 17 segment model [10] and segmental scores assigned as follows: normal − 1, hypokinesia − 2, akinesia − 3, dyskinesia– 4, aneurysmal − 5. WMS was determined by summing the scores for all 17 segments. The Wall Motion Score Index (WMSI) was calculated by dividing the WMS by 17.

- **Thrombus Identification**.

Thrombus was identified on DE-CMR, with features consistent with avascular tissue [12]. The DE-CMR was performed by skilled radiology technicians following a standardized thrombus protocol that has been well studied and validated in previous similar studies [13]. Standard DE-CMR was carried out using an inversion time (TI) principally tailored to null viable myocardium. The thrombus has its unique properties on the standard DE-CMR and contrasts itself from the myocardium [14]. To further enhance the delineation and demarcation of the LVT, additional DE-CMR imaging was conducted using a specified sequence designed to nullify avascular tissue. For cine CMR protocol, LVT identification was purely based on its anatomical appearance [15–17]. In non-contrast 2D TTE, thrombus typically appears as echo-dense and identified by its distinct and clear anatomical appearance [15].

### Sample size

**Fig. 1:**
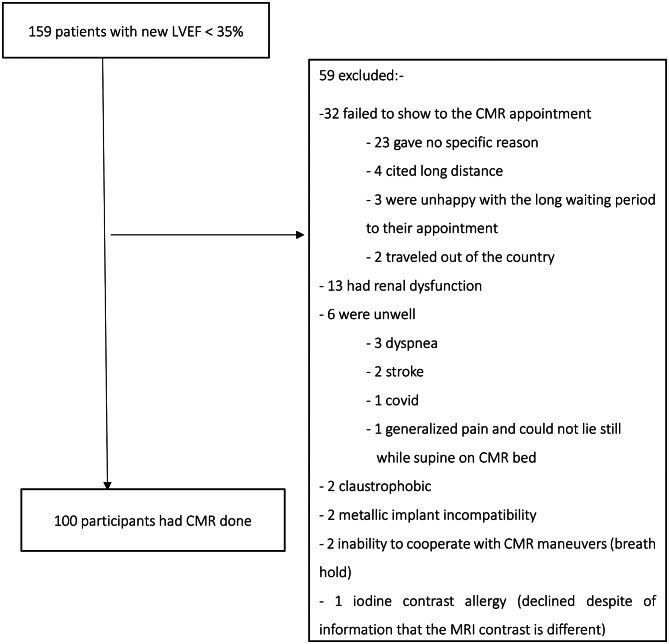
Flow diagram of the study population

The minimal sample size to determine the prevalence of LVT using CMR was estimated at *n* = 100 using the formula below: - (Fig. 1 Below illustrates the flow diagram of the study).

$$n = {{{Z^2}p(1 - p)} \over {{d^2}}}$$

- Where Z is 1.96 for a confidence level of 95%.
- P, the expected CMR prevalence of LVT is 7%. This was based on a CMR study of 784 patients with depressed systolic LV function. [11]
- d2, precision of 5%.

### Statistical analysis

Categorical variables were expressed as frequencies and percentages, and comparisons were made using chi-squared or Fisher’s exact tests. Continuous variables were assessed for normality using the Shapiro-Wilk test and were summarized as means or medians with the respective measures of variability. These variables were compared using the Wilcoxon rank-sum test or Mann-Whitney U test. Participants were categorized into two groups: those with LVT on CMR and those without, and differences between the groups were analyzed. The diagnostic accuracy of two-dimensional echocardiography was determined by calculating sensitivity, specificity, positive predictive value (PPV), negative predictive value (NPV), along with the corresponding 95% confidence intervals. All tests were two-tailed, and p-values < 0.05 were considered statistically significant. Statistical analysis was conducted using SPSS version 24.0 (IBM SPSS Statistics, IBM Corporation, Armonk, NY).

## Ethical clearance

Before obtaining ethical clearance, the study protocol was presented at both the section and departmental levels. It was subsequently approved by the Institutional Scientific and Ethical Review Committee (ISERC) of AKUH, N. The protocol was granted ethical approval with reference number: 2019/IERC-76 [21] by the ISERC of AKUH, N.

Over a twenty-month period, a total of 2,704 echo examinations were done. 540 (20.07%) had a LVEF of less than 35%, with 216 (8%) of these being new systolic heart failure. 159 participants consented to CMR examination. The Flow chart is elaborated in the Fig. 1 below.

## Results

The median age of participants was 58.0 (IQR: 47.0–67.0) years, the majority were male(*n* = 75,75%) and of African origin(*n* = 83,83%). More than half of the patients had Non- ischemic causes of heart failure(*n* = 57,57%), having more than NYHA II symptoms (*n* = 87,87%). A higher percentage with statistical significance (P value < 0.05) of those with LVT had an ischemic etiology of heart failure (*n* = 10, 90.9%). Other baseline and clinical characteristics are described in Table 1 below.

**Table 1:** Demographic and clinical characteristics

Variable	Overall, *N* = 100^1^	Without LVT, *N* = 89	With LVT, *N* = 11	*P*-value^2^
Age (yrs) Median IQR	58.0 (47.0–67.0)	58.0 (47.0–67.0)	60.0 (50.0–65.0)	0.85
Gender, n (%)				0.28
Male	75 (75.0)	65 (73.0)	10 (90.9)	
Ethnicity, n (%)				0.99
Black African	83 (83.0)	74 (83.1)	9 (81.9)	
Asian/Caucasian	17 (17.0)	15 (16.9)	2 (18.2)	
Patient status, n (%)				0.33
Inpatient	39 (39.0)	33 (37.1)	6 (54.5)	
Outpatient	61 (61.0)	56 (62.9)	5 (45.5)	
HTN, n (%)	41 (41.0)	36 (40.4)	5 (45.5)	0.06
DM Type II, n (%)	29 (29.0)	27 (30.3)	2 (18.2)	0.5
Hyperlipidemia, n (%)	20 (20.0)	17 (19.1)	3 (27.3)	0.058
Smoking, n (%)	15 (15.0)	11 (12.4)	4 (36.4)	0.89
Etiology of Heart Failure				
Non- Ischemic	57(57)	56 (62.9)	1(9.1)	
Ischemic	43(43)	33 (37.1)	10 (90.9)	
NYHA Symptoms				0.29
NYHA I	13(13.0)	10 (11.2)	3 (27.3)	
NYHA II	55 (55)	51 (57.3)	4 (36.4)	
NYHA III	28 (28)	24 (27.0)	4 (36.4)	
NYHA IV	4 (4)	4 (4.5)	0 (0)	

^1^n (%); Median (IQR), ^2^Fisher’s exact test; Wilcoxon rank sum test, HTN: Hypertension, DM: Diabetes Mellitus, NYHA: New York Heart Association

The majority of the Cohort had Sinus rhythm on their ECG(*n* = 83,83%), of which only a fraction was in atrial fibrillation (*n* = 12,12%). ECG and laboratory parameters are noted in the Table 2 below.

**Table 2:** ECG and recruitment laboratory parameters of the cohort

	Overall, *N* = 100^1^	Without LVT, *N* = 89	With LVT, *N* = 11	*P*-value^2^
ECG - Variables				0.99
Sinus rhythm, n (%)	83 (83)	72 (80.1)	11 (100)	
Atrial fibrillation, n (%)	12 (12)	12 (13.4)	0	
Atrial flutter, n (%)	3 (3)	3 (3.4)	0	
Ventricular tachycardia, n (%)	2 (2)	2 (2.2)	0	
LBBB, n (%)	22 (22)	20 (22.5)	2 (18.2)	
NTproBNP, median (IQR)	3503 (1896–5680)	2990 (1866–6809)	3647 (1912–5330)	0.99
eGFR, median (IQR)	91.0 (77-101.2)	90.8 (77.0-101.0)	94.9 (87.7-108.3)	0.24

^1^n (%); Median (IQR), ^2^Fisher’s exact test; Wilcoxon rank sum test, LBBB: Left Bundle Branch block, NT pro BNP: N-terminal pro-brain natriuretic peptide, eGFR: Estimated Glomerular Filtration Rate

Table 3 below summarizes chronic Heart Failure (HF) therapy and antithrombotic therapy of the study participants. The most common antiplatelet used was aspirin (*n* = 34, 34%) while B- blockers (*n* = 73,73%), Mineralocorticoid Receptor Antagonists (MRA) (*n* = 78,78%) and Angiotensin Receptor Neprilysin Inhibitors (ARNI) (*n* = 61,61%) were the most routinely used chronic heart failure therapy among the study participants. Table 3 also provides a comparison between those identified with and without LVT on CMR, A higher percentage of patients with LV thrombus(*n* = 7,63.6%) were noted to be on Aspirin when compared to those without LVT (*n* = 27,30%) (P- value 0.042).

**Table 3:** Medications used by the study participants

Chronic antithrombotic therapy, *n* (%)	Overall, *N* = 100^1^	Without LVT, *N* = 89	With LVT, *N* = 11	*P*-value^2^
Aspirin, n (%)	34 (34)	27(30)	7 (63.6)	0.042
Thienopyridines, n (%)	18 (18)	14 (19)	4 (36.3)	0.496
Warfarin, n (%)	5 (5)	3 (3.4)	2 (18.2)	0.071
DOAC, n (%)	14 (14)	9 (10.1)	5 (45.5)	0.300
Parenteral Anticoagulation, n (%)	2 (2)	1 (1.1)	1 (9.1)	0.209
Chronic HF therapy				
ACE-(i)/ARB, n (%)	25 (25)	20 (22.5)	5 (45.5)	0.136
ARNI, n (%)	61 (61)	57 (64)	4 (36.3)	0.103
BB (-), n (%)	73 (73)	64 (72)	9 (81.8)	0.170
MRA, n (%)	78 (78)	71 (79.8)	7 (63.6)	0.252
SGLT2-(i), n (%)	33 (33)	30 (33.7)	3 (27.2)	0.999
Loop diuretic, n (%)	47 (47)	44 (49.4)	3 (27.2)	0.210
Ivabradine, n (%)	5 (5)	5 (5.6)	0 (0)	0.999
Digoxin, n (%)	3 (3)	3 (3.4)	0 (0_	0.999

^1^n (%); Median (IQR), ^2^Fisher’s exact test; DOAC: Direct Oral Anti-Coagulants, ACE-(i): Angiotensin Converting Enzyme Inhibitor, ARB: Angiotensin Receptor Blocker, ARNI: Angiotensin Receptor Neprilysin Inhibitor, BB: B- Blocker, MRA: Mineralocorticoid Receptor Antagonists, SGLT − 2 - (I): Sodium-glucose cotransporter-2 Inhibitor

The prevalence of LVT on DE-CMR was 11%. The median days from 2D TTE to Cardiac MRI was 10 days (IQR:1–12 days). The Median age of those detected with LVT on CMR was 60.0 (50.0–65.0) years, of whom all were male and in sinus rhythm. Majority had an ischemic etiology (*n* = 10,91%) as the cause of their heart failure. Eight participants (73%) had LVT detected on both diagnostic modalities, with most LVT found in the Apex of the LV cavity (*n* = 7, 64%). Majority of those with CMR detected LVT had > 50% transmural involvement of the left ventricle (*n* = 10,91%). The rest of the clinical and imagining characteristics of study participants with LVT is depicted in Table 4 below.

**Table 4:** Clinical and imaging characteristics of participants with LVT

Variable	LVT on CMR, *n* (%) = 11 (11)
Age (yrs), Median IQR	60.0 (50.0–65.0)
Gender	M = 11 (100)
Etiology of HF Ischemic • ACS - Anterior MI • Ischemic Cardiomyopathy on presentation Non- Ischemic Cardiomyopathy	10 (91%) 7 (70) 3 (30) 1(9)
HTN, n (%)	1 (9)
ECG, n (%)	
Sinus rhythm	11 (100)
Atrial fibrillation	0
NT pro BNP (ng/L), median	3647 (IQR 1912–5330)
LVT positive on both CMR and Echo concurrently, n (%)	8 (73)
LVT positive on Echo, absent on CMR, n (%)	0
LVT positive on CMR, absent on Echo, n (%)	3 (27)
Site of LVT, n (%)	
Apical	7 (64)
Apical and septal	3 (27)
Apical aneurysmal	1 (9)
Interval between Echo and CMR, (days)	10 days (IQR:1–12 days)
Anticoagulation, n (%)	9 (81)
% LV with > 50% transmural involvement, n (%)	10 (91)
LV scar on CMR	
Scar, n (%)	10
Scar size as % of LV size	37.6

^1^n (%); Median (IQR), ^2^Fisher’s exact test, LVT: Left Ventricular Thrombus, HTN: Hypertension, ECG: Electrocardiogram, HF: Heart Failure, ACS: Acute Coronary Syndrome, LV: Left Ventricle

Table 5 below illustrates the TTE parameters of the study participants and provides a comparison between those with and without LVT, diagnosed on CMR. The Median LVEF of the study participants was 30% (IQR 20–33). Statistical significance (P- Value < 0.05) was noted when the Left ventricular cavity size and Ejection fraction where compared between those with and without LVT. A Lower LVEF, 19% (IQR 14–31) and greater LV cavity Internal Diameter (LVID) in diastole, 62 mm (IQR 55- 77.5) and systole, 58 mm (IQR 48.5–67) was noted amongst the study participants with LVT.

**Table 5:** Echocardiographic characteristics and comparison according to LVT status on CMR

		CMR LVT Status		
Variable, Median (IQR)	Overall, *N* = 100^1^	Without LVT, *N* = 89	With LVT, *N* = 11	P-value^2^
LVEF (%)	30 (20–33)	30 (20–33)	19 (14–31)	0.002
Anteroseptal wall: Diastole (mm)	8 (6–11)	8 (6–11)	7.1 (6–11)	0.091
Anteroseptal wall: Systole (mm)	9 (7–14)	10 (8–14)	8 (7-13.5)	0.099
Inferolateral wall: Diastole (mm)	9 (8–12)	9 (8–12)	8.8 (7.5–10)	0.15
Inferolateral wall: Systole (mm)	10 (9–13)	11 (8–13)	11 (8 − 1)	0.24
LVID: Diastole (mm)	55 (51-62.2)	55.6 (51–61)	62 (55.5–77.5)	0.017
LVID: Systole (mm)	47 (42-53.1)	47.2 (42–50)	58 (48.5–67)	0.009
LV mass (gm)	129.5 (113.9-148.3)	128 (113-147.9)	123.0 (120.2-169.4)	0.31
LV mass index (gm/M^2^)	69.8 (62-74.5)	69.8 (61.3–74.5)	67 (61.1–72)	0.27
End Diastolic Volume (ml)	140.5 (105-171.5)	139 (102–170)	159 (137–212)	0.13
End Systolic Volume (ml)	98.5 (65-134.5)	97 (62–128)	120 (95–176)	0.14
Stroke volume (ml/beat)	38 (28-45.2)	38 (29–45)	36 (25-47.5)	0.76
Stroke volume index (ml/beat/M2)	20.4 (15.6–24.6)	20.4 (16-24.5)	21 (13-25.3)	0.75
Cardiac Output (L/min.)	3.160 (2.397–4132)	3.200 (2400–4200)	2.890 (2065–3840)	0.17
Cardiac Index (L/min./M^2^)	1.731 (1316–2202)	1.762 (1336–2240)	1.517 (990–1975)	0.22
LA size (cm)	4.1 (3.7–4.6)	4.1 (3.7–4.6)	4.4 (3.9–4.6)	0.37
LA volume index (ml/M^2^)	34.2 (30.3–38.3)	34.2 (30.2–38.0)	35.5 (33.2–39.2)	0.27
E/A	1.6 (0.9–2.4)	1.5 (0.8–2.4)	2.0 (1.3–3.8)	0.071
E/e’	13.6 (10-16.4)	13.5 (10-16.3)	15.3 (10.8–17.7)	0.15
RVSP	40.3 (35-48.1)	40 (35–46)	43 (38.4–50)	0.29
TAPSE (cm)	1.8 (1.7–1.9)	1.8 (1.7-2.0)	1.6 (1.6–1.8)	0.074

^1^n (%); Median (IQR), ^2^Fisher’s exact test, ^2^Wilcoxon rank sum test, LVEF: Left Ventricular Ejection Fraction, LVID: Left Ventricular internal Diameter, LV: Left Ventricle, LA: Left atrium, RVSP: Right Ventricular Systolic Pressure, TAPSE: Tricuspid Annular Plane Systolic Excursion

CMR characteristics for the cohort are presented in Table 6 below. Cine-CMR revealed an overall median LVEF of 33% and Right Ventricular Ejection Fraction (RVEF) of 59%. When the two groups were compared, the Anterior Wall thickness (AWT) was much smaller in those study participants detected with LVT, 6 mm (3.8–7.5) than those without LVT 8 mm [6–10] (P-value 0.037). Other comparative findings are well portrayed in Table 6 below.

**Table 6:** Cardiac MRI characteristics and comparison according to LVT status on CMR

Variable, Median (IQR)	Overall, *N* = 100^1^	Without LVT, *N* = 89	With LVT, *N* = 11	*P*-value^2^
LVEF (%)	33 (23-40.2)	33 (23–40)	31 (13–44)	0.59
LV AWT: Diastole (mm)	8 (6-9.5)	8 (6–10)	6 (3.8–7.5)	0.037
LV AWT: Systole (mm)	10 (7–12)	10 (8–12)	7 (4.5-9)	0.026
LV PWT: Diastole (mm)	8 (6–9)	8 (6–9)	7 (6.2–8.8)	0.06
LV PWT: Systole (mm)	10 (8–12)	10 (8–12)	9.4 (8–11)	0.071
LVID: Diastole (mm)	59 (52-72.8)	59 (52-61.5)	62 (57–69)	0.083
LVID: Systole (mm)	45 (40–63)	45 (41-48.5) 50	50 (54–63)	0.11
LV mass (gm)	119 (87.8-149.2)	109 (70–116)	121 (97–155)	0.12
LV mass index (gm/M^2^)	60.5 (47.9–77.4)	60.3 (37.8–64)	63 (43.5–79.5)	0.52
LV End Diastolic Volume (ml)	145 (131-258.2)	141 (136–179)	158 (133–259)	0.060
LV End Systolic Volume (ml)	84 (77.2-187.5)	79.7 (65.5–131)	105 (91–189)	0.08
LV Stroke volume (ml/beat)	61 (51-80.4)	62 (51-79.5)	53 (47-75.5)	0.26
LV Stroke volume index (ml/beat/M^2^)	31.9 (27.8–42)	32 (28–42)	29.4 (23.9–39)	0.20
LV Cardiac Output (L/min.)	4.8 (3.8–5.5)	4.8 (4-5.5)	3.7 (3.3–5.2)	0.085
LV Cardiac Index (L/min./M^2^)	2.4 (2–3)	2.5 (2–3)	2 (1.8–2.6)	0.051
RVEF (%)	59 (35-55.2)	57 (30.5–63.5)	45 (36–55)	0.43
RV End Diastolic Volume (ml)	134 (115.8-181.2)	129 (102–141)	146 (118–183)	0.11
RV End Systolic Volume (ml)	51 (41–111)	51 (42-78.5)	81 (53–115)	0.067
RV Stroke Volume (ml/beat)	63 (52–79)	63 (53–78)	60 (56.5–83)	0.51
RV Stroke Volume index (ml/beat/M^2^)	34.2 (27-42.4)	34.7 (28.4–42.2)	31.6 (24.9–43.1)	0.55
RV Cardiac Output (L/min.)	5.6 (3.9–5.8)	5.6 (3.9–5.9)	4.9 (4-5.8)	0.59
RV Cardiac Output Index (ml/beat/M^2^)	2.5 (2.1-3.0)	2.5 (2.1-3.0)	2.3 (2.1–2.9)	0.39

n (%); Median (IQR), ^2^Fisher’s exact test, ^2^Wilcoxon rank sum test, LVEF: Left Ventricular Ejection Fraction, AWT: Anterior wall Thickness, PWT: Posterior Wall Thickness, LV: Left Ventricle, RVEF: Right Ventricle Ejection Fraction

Table 7. below highlights the diagnostic accuracy of the TTE to identify LVT when compared to CMR in the study population. In total There were 27 study participants classified as possibly having LVT on TTE, where only 11 of them were confirmed on CMR. Of the 27 participants with LVT on Echo, 21 (77.7%) were, commenced on anticoagulation for presumed LVT. These participants continued anticoagulation therapy from the time of recruitment to the time to CMR. The Diagnostic accuracy for the TTE was calculated to be 0.78 as seen below.

**Table 7:** Diagnostic accuracy of TTE

Measure	Estimate
Sensitivity	0.72
Specificity	0.78
Positive predictive value	0.29
Negative predictive value	0.98
LRT+	3.30
LRT-	0.34
Diagnostic accuracy	0.78

LRT: Likelihood Ratio test

**Fig. 2:**
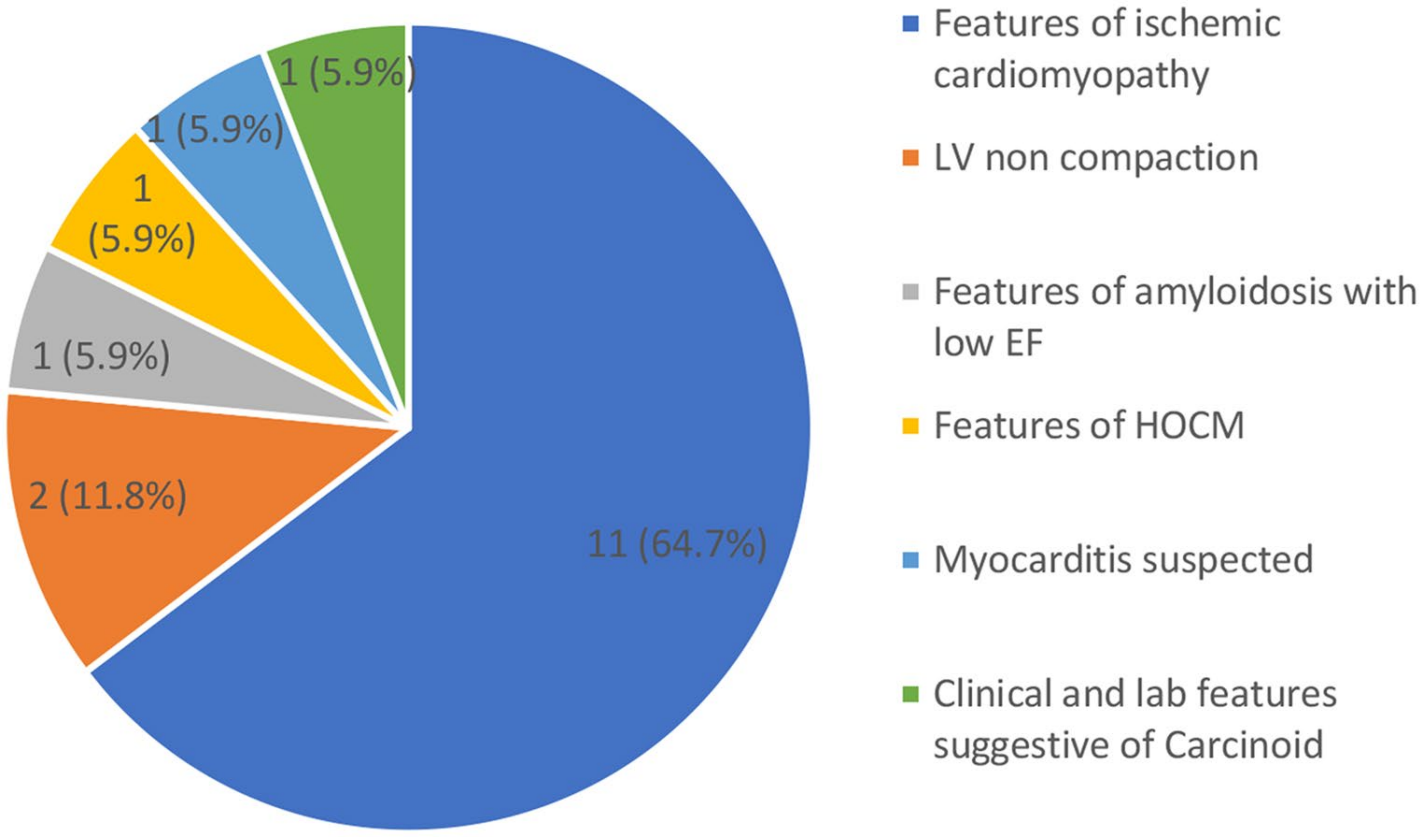
Etiology of heart failure determined by CMR, where etiology was previously unknown

CMR was able to further establish the etiology of HF in 17 participants as illustrated in Fig. 2 below. Additionally, CMR was also helpful in excluding hemochromatosis in one patient in whom serum transferrin saturations levels were mildly elevated, with HFE testing being indeterminate. CMR did not show features suggestive of hemochromatosis.

## Discussion

To the best of our knowledge, this is the largest prospective cohort study in the region evaluating CMR in patients with moderate to severely reduced LV systolic function, with corresponding TTE data for comparison. The overall incidence of LVT was 11%, predominantly in patients with ischemic cardiomyopathy following AMI. Reported incidence of LVT varies globally, typically ranging between 4% and 14% across diverse populations [8, 12, 16, 17]. While exploring the underlying reasons for the similarities and disparities was beyond this study scope, differences in geographical location [18], healthcare infrastructure [18], diagnostic methods [8, 18], demographic [19], and risk factor prevalence [20] are well known factors for possible disparities. Notably, our findings are consistent with previous studies, which also identified ischemic heart failure and increased left ventricular internal diameter in diastole (LVIDd) as factors associated with LVT formation.

DE- CMR is currently the gold standard imaging technique for LVT detection. our study findings demonstrated that non-contrast 2D TTE shows a reasonable sensitivity (0.72) and specificity (0.78) for detecting LVT when compared to CMR. The negative predictive value (0.98) of 2D TTE was notably excellent, indicating that it is highly effective in ruling out LVT in patients with heart failure. These results are consistent with various similar studies done globally [9, 19–21]. Our study identified a trend in which TTE overestimated the presence of LVT, with 27% of the cases showing its presence, but only 11% being confirmed by CMR. This highlights discrepancies and a temporal disconnect between the two diagnostic methods. In addition to DE-CMR’s superior spatial and temporal resolution, the differences in LVT detection rates between TTE and CMR can be attributed to several factors. One key factor is anticoagulation therapy [22], which may reduce the presence of LVT between the initial echocardiogram and the follow-up CMR. Despite performing CMR within 14 days of the 2D TTE, our institution conducted CMR on Tuesdays and Thursdays, so anticoagulation therapy might have led to a reduction in the thrombus presence between the echocardiogram and CMR. A clot visible on TTE could have disappeared by the time of the CMR due to changes induced by anticoagulation, potentially resulting in a false negative result. In hindsight, the timing [23] of the initial TTE is another crucial factor. If the TTE is performed too early during the acute phase of heart failure or shortly after a myocardial infarction, thrombus detection may be overlooked, as the thrombi may not yet have formed or grown large enough to be detected. Echocardiographic limitations [9] can also contribute to the disparity. Visualizing the LV apex can be difficult, with structures such as trabeculae or near-field artifacts sometimes mimicking LVT, leading to potential overestimation of its presence. In situations of uncertainty, clinicians may tend to consider the possibility of LVT, as anticoagulation for stroke prevention is generally regarded as safer than the risk of a stroke. This can result in overreporting the presence of LVT on TTE. These factors emphasize the complexity of diagnosing LVT and highlight the importance of carefully considering timing, anticoagulation status, imaging modality, and other technical factors in clinical practice.

our results provide insights into the use of routine clinical TTE as a screening tool for LV thrombus. The findings contribute to the growing body of literature supporting the utility of DE CMR for assessing LVT in high-risk populations, including patients with advanced LV systolic dysfunction, LV aneurysms, or large myocardial infarctions.

## Limitations

This study should be interpreted in light of several limitations. First, it was conducted at a single urban referral and specialist care center in Kenya, which may limit the generalizability of the findings to rural populations or other countries in the region. Nevertheless, as a national referral center, the patient cohort likely captures a diverse cross-section of individuals with heart failure across the country. Second, we did not use echocardiographic contrast, which could have enhanced the sensitivity of TTE in detecting LVT, potentially improving diagnostic accuracy. Additionally, patients with severe renal dysfunction (eGFR < 30 ml/min/1.73 m²), gadolinium allergy, or non-MRI-compatible implants were excluded—common limitations in contrast-enhanced CMR studies that may introduce selection bias. The study period overlapped with the peak of the COVID-19 pandemic, which likely influenced healthcare-seeking behavior and may have skewed the prevalence estimates of LVT. Furthermore, participants with suspected thrombus on echocardiography may have been more inclined to enroll, contributing to additional selection bias. Finally, although TTE demonstrated good negative predictive value, the study design did not allow us to explore clinical or demographic factors associated with LVT formation. A larger, multicenter study with a different design may be needed to identify such associations.

## Conclusion

In conclusion, this study provides important assessments. 11% of patients were identified as having LVT on CMR. Our findings indicate that 2D echocardiography provides reasonable sensitivity and specificity for detecting LVT, with an excellent negative predictive value, making it a useful tool for ruling out LVT in clinical practice. Ischemic heart failure and the presence of a dilated LV cavity remain significant contributors to thrombus formation. While our results support the use of routine echocardiography as a screening tool, further research, including multi-center studies and the validation of CMR protocols, is necessary to refine diagnostic strategies and enhance patient management.

### Future prospective

A dedicated DE-CMR protocol, incorporating long-T1 imaging, could serve as an effective tool to confirm an LVT diagnosis, particularly when routine echocardiography results are inconclusive or when sonographic contrast agents are unavailable. However, while these results are promising, further validation by other research groups is needed before recommending widespread changes in clinical practice.

## Data Availability

No datasets were generated or analysed during the current study.
